# Association of inflammatory markers and hematological inflammatory indices in predicting pseudoexfoliation

**DOI:** 10.6026/973206300212405

**Published:** 2025-08-31

**Authors:** Harshitha C., Chaitra M.C.

**Affiliations:** 1 Department of Ophthalmology, Sri Devaraj Urs Medical College, R.L. Jalappa Hospital, Kolar, Karnataka, India

**Keywords:** Pseudoexfoliation syndrome, inflammation, Neutrophil -Lymphocyte Ratio (NLR), systemic immune-Inflammation index (SII), systemic markers, Red Cell width distribution (RDW)

## Abstract

Pseudoexfoliation syndrome (PEX) is a common age-related ocular disorder with systemic inflammatory implications. This cross-sectional study investigated the role of hematological indices-NLR, PLR, dNLR, RDW, SII, SIRI and PIV-in 195 PEX patients and 195 controls. All markers, including novel indices like SII and SIRI, were significantly elevated in PEX (p < 0.05). RDW was also higher, indicating oxidative stress and systemic involvement. These indices may serve as accessible, cost-effective predictors of PEX-related inflammation.

## Background:

Pseudoexfoliation syndrome (PEX) is a clinical condition characterized by the accumulation of whitish-grey fibrillar extracellular material in the anterior chamber of the eye [1]. It is considered a common age-related systemic disorder, affecting approximately 10% to 20% of the population over the age of 60 and involves both genetic and non-genetic factors in its pathogenesis [2]. The prevalence of PEX among patients undergoing cataract surgery varies widely-from 5% in South Africa to 16% in Turkey and up to 39% in Ethiopia [3]. PEX is associated with increased intraocular pressure and intraoperative complications such as poorly dilating pupils and zonular instability. The accumulation of exfoliative material obstructs trabecular meshwork outflow, leading to pseudoexfoliative glaucoma-a more aggressive form of secondary open-angle glaucoma [4]. High levels of neutrophils and the neutrophil-to-lymphocyte ratio (NLR) are among the most widely studied markers of systemic inflammation, with NLR reflecting both inflammation and physiological stress due to its incorporation of lymphopenia. Additionally, the platelet-to-lymphocyte ratio (PLR) has been explored as a biomarker for inflammation, particularly in oncological and cardiovascular conditions [5]. Red cell distribution width (RDW), which reflects variability in erythrocyte size, has been found to correlate with poor prognosis in various cardiovascular and cerebrovascular diseases [6]. More recently, composite inflammatory markers such as the Systemic Inflammation Response Index (SIRI) and the Prognostic Inflammation Value (PIV) have been introduced. These indices are considered robust tools for assessing systemic immune-inflammatory status and are increasingly used to evaluate prognosis in conditions like cancer, acute coronary syndrome and sepsis [7, 8]. Therefore, it is of clinical interest to investigate the association of these indices with PEX and explore their potential as predictors of inflammatory status in affected individuals.

## Objectives:

[1] To determine the Neutrophil-Lymphocyte Ratio (NLR), derived NLR and platelet -Lymphocyte Ratio (PLR) and compare them in Pseudoexfoliation group and controls.

[2] To determine systemic immune-Inflammation index (SII), Systemic Inflammation Response Index (SIRI) and Prognostic Inflammation value (PIV) in Pseudoexfoliation patients.

[3] To determine the association of Red Cell width distribution in Pseudoexfoliation patients.

## Materials and Methods:

## Source of data:

This observational cross-sectional study, conducted over one year, involved 390 participants aged >50 years from the Ophthalmology Outpatient Department at R. L. Jalappa Hospital, Kolar, affiliated with Sri Devaraj Urs Medical College, Kolar from 2023 to 2024, after obtaining ethical clearance from Institutional Ethical Committee and written informed consent from the subjects. Of these, 195 had Pseudoexfoliation (PEX) and 195 were healthy controls.

## Inclusion criteria:

Both male and female patients > 50 years with bilateral Pseudoexfoliation as cases and age and gender matched healthy individuals without PEX as controls.

## Exclusion criteria:

Systemic infectious diseases, Cardiovascular diseases, Autoimmune disorders, Malignancies, chronic kidney and liver failure, Asthma, Rheumatologic diseases, Hematologic diseases, Any history of surgery within the past three months, Individuals with chronic or recurrent inflammatory eye conditions, Glaucoma, Ocular injuries.

## Methodology:

After taking written and informed consent, all the patients admitted in Ophthalmology department at R. L Jalappa Hospital and Research Centre, attached to Sri Devaraj Urs Medical College who meet inclusion criteria are recruited for the study. Patient's demographic details will be noted. Following the clinical examination which includes Visual acuity by Snellens chart for distant vision and near vision by Jaegers chart, Refraction, Intra ocular Pressure by Goldman Applanation Tonometer, Anterior segment by slit-lamp and Fundus evaluation by direct and indirect ophthalmoscope, the patients were classified into two groups; the PEX group and the non-PEX group. Diagnosis of PEX is done by standardized clinical examination for signs of the syndrome by slit lamp.

## Diagnostic criteria of PEX:

Characteristic grayish-white exfoliative material on the anterior capsule and/or pupillary margin in mydriatic pupil by slit lamp. Also, typical flakes on the iris surface, in either eye were considered as a diagnostic parameter. Blood samples of all participants were taken from all participants and examination includes evaluating hemogram parameters. [Neutrophil to Lymphocyte Ratio (NLR), Platelet to Lymphocyte Ratio (PLR)]. The calculation of derived NLR (dNLR) is done by dividing the absolute neutrophil count by the absolute white blood cell (WBC) count minus the absolute neutrophil count. Systemic immune-Inflammation index (SII) will be calculated by multiplying the neutrophil count by the platelet count, then dividing by the lymphocyte count. Systemic Inflammation Response Index (SIRI) is calculated by multiplying the neutrophil count by the monocyte count and then dividing by the lymphocyte count. Conversely, Prognostic Inflammation Value (PIV) is determined by multiplying the neutrophil count, the monocyte count and the platelet count, then dividing by the lymphocyte count. Using these formulas, SIRI, PIV and other indicators will be computed in Excel and the data will be organized for analysis.

## Statistical analysis:

Data will be entered into Microsoft excel data sheet and will be analyzed using SPSS 22 version software. Categorical data will be represented in the form of Frequencies and proportions. Chi-square will be used as test of significance. Continuous data will be represented as mean and standard deviation. Independent t test will be used as test of significance to identify the mean difference.

## Graphical representation of data:

MS Excel and MS word was used to obtain various types of graphs P value (Probability that the result is true) of <0.05 was considered as statistically significant after assuming all the rules of statistical tests.

## Results:

This study evaluated 195 PEX patients and 195 healthy controls, analyzing their demographic characteristics, hematological parameters and inflammatory indices to explore the role of systemic inflammation in pseudoexfoliation syndrome (PEX). The results confirm significant differences between the two groups, reinforcing the inflammatory nature of PEX. The mean age of PEX patients and controls was similar, ensuring no age-related bias and the gender distribution was also statistically comparable, maintaining balance between groups (Table 1 - see PDF).

Neutrophils were significantly elevated in PEX patients, indicating increased systemic inflammation. Lymphocyte levels were reduced, further supporting immune dysregulation in PEX. Platelet counts were higher in PEX patients, suggesting a hypercoagulable state associated with systemic inflammation (Table 2 - see PDF). NLR, a widely recognized marker of inflammation, was significantly higher in PEX patients, consistent with previous studies linking elevated NLR to ocular and systemic inflammation. PLR was also increased in PEX patients, suggesting platelet activation, which is commonly associated with vascular dysfunction and chronic inflammation. dNLR, an alternative form of NLR, was similarly elevated, reinforcing the role of neutrophil-driven inflammation in PEX pathophysiology (Table 3 - see PDF). SII, an emerging inflammatory biomarker, was significantly higher in PEX patients, indicating a stronger systemic inflammatory response. SIRI was elevated in the PEX group, emphasizing the role of monocytes in chronic inflammation. PIV which combines neutrophil, monocyte and platelet counts was also higher, further validating the inflammatory hypothesis in PEX (Table 4 - see PDF). DW, a marker of oxidative stress and vascular dysfunction, was significantly higher in PEX patients. This suggests that PEX is not only an ocular disorder but also has systemic implications, particularly in vascular and cardiovascular health (Table 5 - see PDF). The overall WBC count did not show a significant difference (p = 0.238) between PEX patients and controls. However, individual WBC components (neutrophils, lymphocytes) showed significant differences, suggesting that the immune response in PEX is more specific rather than a general leukocytosis (increase in WBC count) (Table 6 - see PDF). No significant difference in hemoglobin levels (p = 0.327) suggests that PEX does not cause or result from anemia. This reinforces that PEX is primarily an inflammatory condition rather than a hematological disorder affecting oxygen transport (Table 6 - see PDF). MPV, an indicator of platelet activation, was similar in both groups (p = 0.451). Although PEX patients had a higher platelet count, their platelet volume remained unchanged, suggesting that platelet function, rather than size, may be more relevant to inflammation in PEX (Table 6 - see PDF). The total RBC count did not differ significantly between PEX and control groups (p = 0.521), indicating that PEX does not affect erythropoiesis (red blood cell production). This further supports the idea that PEX is mainly an inflammatory disease rather than one affecting red blood cell physiology (Table 6 - see PDF).

## Discussion:

The findings of this study confirm that pseudoexfoliation syndrome (PEX) is associated with a systemic inflammatory response, as evidenced by significantly elevated inflammatory indices, including Neutrophil-to-Lymphocyte Ratio (NLR), Platelet-to-Lymphocyte Ratio (PLR), Derived NLR (dNLR), Systemic Immune-Inflammation Index (SII), Systemic Inflammation Response Index (SIRI) and Prognostic Inflammation Value (PIV). These results align with previous studies that have identified a strong inflammatory component in PEX pathogenesis. A study by Ozgonul *et al.* (2016) [9] found that NLR and PLR were significantly higher in PEX and pseudoexfoliation glaucoma (PEXG) patients than in controls, supporting our findings that inflammatory markers play a crucial role in PEX progression. Similarly, Bashir *et al.* (2022) [2] reported elevated Red Cell Distribution Width (RDW) levels in PEX patients, which correlate with our study, where RDW was significantly higher in the PEX group. This suggests that oxidative stress and vascular dysfunction may be key contributors to PEX development, reinforcing the idea that PEX is not only an ocular disease but also has systemic implications. In our study, advanced inflammatory indices (SII, SIRI and PIV) were significantly elevated in PEX patients, providing additional insight into the immune response associated with the disease. Elbeyli *et al.* (2022) [10] and Ozarslan *et al.* (2022) [11] have previously reported that SII is an effective biomarker in ocular inflammatory diseases such as diabetic macular edema and dry eye disease, further supporting our findings that SII can be a useful indicator of systemic inflammation in PEX. The elevated SIRI values in PEX patients highlight the role of monocytes in chronic inflammation, a factor that has also been observed in other systemic inflammatory disorders. While the majority of hematological parameters showed significant differences, some markers, such as total white blood cell (WBC) count, hemoglobin levels and mean platelet volume (MPV), did not exhibit statistically significant differences between the PEX and control groups.

This is consistent with findings from Yilmaz and Yilmaz (2025) [5], who observed that although NLR and PLR were significantly elevated in PEX and PEXG patients, total WBC count and hemoglobin levels did not differ significantly from controls, suggesting selective rather than generalized inflammatory activation. This suggests that PEX is characterized by specific immune responses rather than a general leukocytosis. Similarly, the lack of significant difference in hemoglobin levels suggests that PEX does not directly cause anemia, reinforcing the idea that its pathogenesis is primarily driven by inflammatory and oxidative stress mechanisms rather than hematological abnormalities. The findings from this study also provide evidence that PEX may have cardiovascular implications, as previously suggested by studies linking elevated RDW to cardiovascular diseases. The significant increase in RDW in PEX patients aligns with research by Bengi *et al.* (2018) [12], which demonstrated that PEX patients had a higher risk of vascular dysfunction, possibly due to systemic inflammation and oxidative stress. Given this association, routine cardiovascular evaluation in PEX patients may be beneficial for early identification of comorbidities. Overall, our results support the growing body of evidence indicating that PEX is not just an isolated ocular disorder but a systemic inflammatory condition with potential vascular and cardiovascular implications. The significant elevation of inflammatory markers in PEX patients highlights the need for further research into targeted anti-inflammatory interventions that may help slow disease progression and reduce the risk of associated complications such as glaucoma and cardiovascular diseases.

## Conclusion:

PEX is significantly associated with elevated systemic inflammatory markers, including NLR, PLR, dNLR, SII, SIRI, PIV, and RDW. These indices offer a simple, cost-effective means for early diagnosis and risk assessment in PEX. Their use may enhance clinical decision-making, especially in detecting systemic involvement and monitoring progression.
